# Misregulation of DNA damage repair pathways in HPV-positive head and neck squamous cell carcinoma contributes to cellular radiosensitivity

**DOI:** 10.18632/oncotarget.16265

**Published:** 2017-03-16

**Authors:** Catherine M. Nickson, Parisa Moori, Rachel J. Carter, Carlos P. Rubbi, Jason L. Parsons

**Affiliations:** ^1^ Cancer Research Centre, Department of Molecular and Clinical Cancer Medicine, University of Liverpool, Liverpool L3 9TA, UK

**Keywords:** HPV, HNSCC, DNA repair, base excision repair, PARP

## Abstract

Patients with human papillomavirus type 16 (HPV)-associated oropharyngeal squamous cell carcinomas (OPSCC) display increased sensitivity to radiotherapy and improved survival rates in comparison to HPV-negative forms of the disease. However the cellular mechanisms responsible for this characteristic difference are unclear. Here, we have investigated the contribution of DNA damage repair pathways to the *in vitro* radiosensitivity of OPSCC cell lines. We demonstrate that two HPV-positive OPSCC cells are indeed more radiosensitive than two HPV-negative OPSCC cells, which correlates with reduced efficiency for the repair of ionising radiation (IR)-induced DNA double strand breaks (DSB). Interestingly, we show that HPV-positive OPSCC cells consequently have upregulated levels of the proteins XRCC1, DNA polymerase β, PNKP and PARP-1 which are involved in base excision repair (BER) and single strand break (SSB) repair. This translates to an increased capacity and efficiency for the repair of DNA base damage and SSBs in these cells. In addition, we demonstrate that HPV-positive but interestingly more so HPV-negative OPSCC display increased radiosensitivity in combination with the PARP inhibitor olaparib. This suggests that PARP inhibition in combination with radiotherapy may be an effective treatment for both forms of OPSCC, particularly for HPV-negative OPSCC which is relatively radioresistant.

## INTRODUCTION

Over half a million new cases of head and neck squamous cell carcinoma (HNSCC) are reported per year, and particularly over the last three decades there has been a rapid rise in the incidence of human papillomavirus type-16 (HPV)-associated cancers of the oropharynx [1, 2]. Interestingly, studies have shown that patients with HPV-positive oropharyngeal squamous cell carcinoma (OPSCC) have improved survival rates in comparison to their HPV-negative counterparts [3–6]. This is despite the fact that HPV-positive cancers typically present with clinicopathological features (ie. nodal metastasis and extracapular spread) that are considered strong prognosticators of poor outcome in HPV-negative cancers. Furthermore, HPV-positive OPSCC are more sensitive to radiotherapy and chemotherapy than HPV-negative tumours which contributes to the improved prognosis [7]. Importantly, cultured cells derived from patients with HPV-positive and HPV-negative OPSCC recapitulate the same characteristic difference in radiosensitivity than that of the original tumour [8–10]. HPV infection causes expression of the E6 and E7 oncogenes, leading to multiple cellular effects including degradation of the tumour suppressor proteins p53 and Rb that control cell cycle progression and are involved in the DNA damage response [11]. Nevertheless, the underlying cellular mechanisms responsible for the apparent increased sensitivity of HPV-positive cancers to radiotherapy (ionising radiation; IR) are unclear.

It has been suggested, using HNSCC cell lines as models that the degree of radiosensitivity correlates with the effectiveness of signalling through the Akt protein kinase. Therefore an absence of Akt activation, as observed in the HPV-positive UCPI-SCC090 cell line, caused severe radiosensitivity in comparison to HPV-negative SQ20B cells where Akt-dependent phosphorylation is fully functional [8]. More recently however, it has also been proposed that HPV-positive HNSCC cells (particularly UD-2, UMSCC47 and UPCI-154) are more radiosensitive than HPV-negative cells due to an impairment in the repair of DNA double strand breaks (DSBs) and an extensive G2 cell cycle arrest [9]. Indeed, residual DSBs measured via phosphorylated histone H2AX/p53-binding protein 1 (γH2AX/53BP1) foci in HPV-positive cells were found to persist 24 h post-IR [9]. In support of this IR-induced DSBs measured indirectly by γH2AX foci, but also directly by neutral comet assays, do persist in HPV-positive (UMSCC47 and UPCI-SCC154) versus HPV-negative (UMSCC1) cells [12]. This defect in DSB repair was suggested to be a consequence of reduced expression of both non-homologous end joining (NHEJ) proteins (53BP1 and DNA-dependent protein kinase; DNA-Pk) as well as homologous recombination (HR) proteins (breast cancer 2; BRCA2 and RAD51). Indeed reduced DNA-Pk, BRCA2 and RAD51 foci in HPV-positive cells at various time points post-IR were observed, but interestingly there was no decrease in 53BP1 foci in these cells. Nevertheless with these limited studies, further characterisation of HNSCC cells is necessary to further understand the molecular basis behind the inherent difference in radiosensitivity between HPV-positive and HPV-negative HNSCC cells.

In addition to DSBs, IR also induces DNA base lesions, DNA base loss (apurinic/apyrimidinic or AP sites), and DNA single strand breaks (SSB). This large proportion (> 90 %) of IR-induced DNA damage is repaired by the base excision repair (BER) pathway [13, 14]. The importance of BER in the cellular DNA damage response to IR has been shown using cells derived from BER-deficient mice or using siRNA-mediated knockdowns of key BER proteins. For example, cells lacking X-ray cross-complementing protein-1 (XRCC1), DNA polymerase β (Pol β), AP endonuclease-1 (APE1) and polynucleotide kinase phosphatase (PNKP) display increased cellular radiosensitivity [15–18]. This suggests that the ability of radiotherapy to kill cancer cells is partially dependent on the levels of BER proteins, and thus their cellular BER capacity. Interestingly, BER protein levels are frequently misregulated in several human cancers [19, 20] suggesting that BER is crucial for genome stability and cancer prevention, but also that BER misregulation may have important consequences for the responsiveness of these tumours to radiotherapy and chemotherapy. In HNSCC, there is a growing body of evidence, albeit conflicting, suggesting that BER mRNA and protein levels are altered in cells and tissues derived from these patients, and that this correlates with outcome and response to therapy. Downregulation of gene expression of XRCC1 and the 8-oxoguanine DNA glycosylase (OGG1) has been observed in Pakistani and North Indian HNSCC patients [21–23]. Similarly, reduced XRCC1 protein expression has been found in laryngeal cancer which correlated with the increased sensitivity to radiotherapy [24]. In contrast, high XRCC1 protein expression in HNSCC patients correlates with poorer survival particularly to those that received chemoradiation [25]. Upregulation of APE1 protein expression has been observed in HNSCC tissues [22], and has been linked to resistance to chemoradiation and poorer survival [26]. Finally, poly(ADP-ribose) polymerase 1 (PARP-1) is overexpressed in nasopharyngeal carcinoma cells and tissues, and PARP inhibitors radiosensitise cells to IR whilst reducing tumour growth in combination with IR in xenograft models [27]. Despite this evidence, a role for HPV, specifically in OPSCC, in modulation of BER protein levels and the correlation with radiosensitivity has not previously been reported.

Cumulatively these data suggest that DNA repair pathways, including DSB and BER, may play important roles in both the development of HNSCC, but also in the response of these tumours to radiotherapy. Here we report that cells derived from HPV-positive OPSCC are indeed more radiosensitive than HPV-negative cells, which in turn correlates with defective IR-induced DSB repair. Surprisingly however, we also demonstrate that expression of key BER and SSB repair proteins, including XRCC1, Pol β, PNKP and PARP-1, are elevated in HPV-positive OPSCC cells, which increases the capacity of these cells to perform BER/SSB repair. Interestingly we further discovered that HPV-positive, but more so HPV-negative OPSCC cells exhibit increased radiosensitivity following PARP inhibition. This suggests that this therapeutic strategy could be exploited for both forms of the disease particularly for HPV-negative OPSCC which is relatively radioresistant.

## RESULTS

### HPV-positive OPSCC cells are radiosensitive and display reduced efficiencies of DNA DSB repair

**Figure 1:**
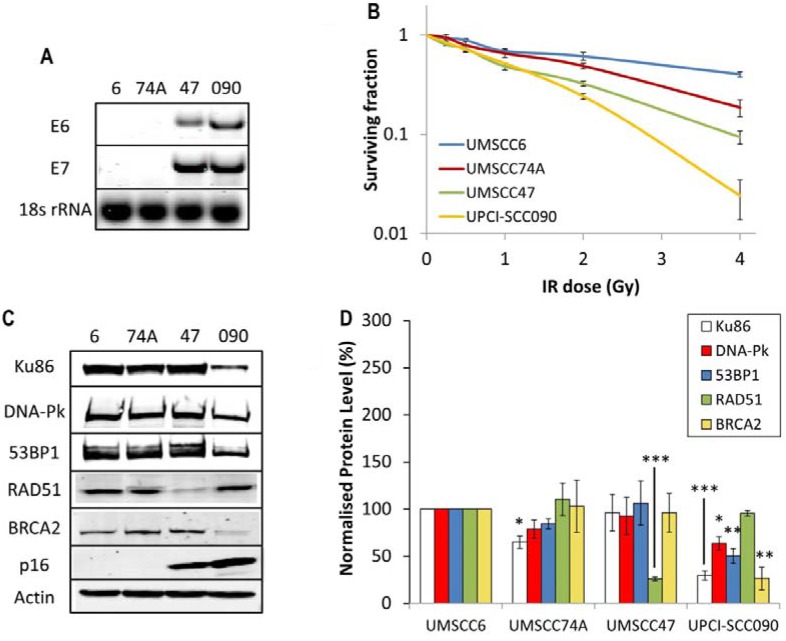
Analysis of radiosensitivity of HPV-negative and HPV-positive OPSCC cells and correlation with DSB repair protein levels (**A**) RT-PCR of cDNA prepared from OPSCC cells confirming HPV status through expression of E6 and E7 oncogenes, in comparison to 18s rRNA as a control, as analysed by agarose gel electrophoresis. (**B**) Clonogenic survival of OPSCC cells was analysed following treatment with increasing doses of x-ray irradiation (0–4 Gy). Shown is the surviving fraction with standard errors from at least three independent experiments. A comparison of the surviving fraction at 2 Gy (SF2) by one-way ANOVA reveals *p* < 0.01 (UMSCC6 vs UMSCC47), *p* < 0.005 (UMSCC6 vs UPCI-SCC090), *p* < 0.02 (UMSCC74A vs UMSCC47) and *p* < 0.002 (UMSCC74A vs UPCI-SCC090). (**C**) Whole cell extracts from OPSCC cells were prepared and analysed by 10 % or 6 % SDS-PAGE and immunoblotting with the indicated antibodies. (**D**) Levels of DSB repair proteins relative to actin were quantified from at least three independent experiments. Shown is the mean protein level relative to actin with standard errors from at least three independent experiments, normalised to those calculated in the HPV-negative UMSCC6 cell extracts which was set to 100 %. *p < 0.05, **p < 0.02, ***p < 0.005 as analysed by a one sample *t*-test of normalised protein levels in the respective cell extracts relative to the UMSCC6 extracts.

It has previously been shown that the apparent increased radiosensitivity of HPV-positive HNSCC in comparison to HPV-negative HNSCC can be recapitulated in immortalised cell lines derived from the respective tumours [8–10]. This suggests that these cell lines are a good *in vitro* model for investigating the molecular and cellular mechanisms determining the radiobiology of HNSCC. Using specifically OPSCC cell lines, where expression of E6 and E7 oncogenes were confirmed (Figure 1A), we were indeed able to reproduce the statistically significant increased radiosensitivity of two HPV-positive OPSCC cell lines (UMSCC47 and UPCI-SCC090) in comparison to two HPV-negative OPSCC cell lines (UMSCC6 and UMSCC74A; Figure 1B). As previously reported, there is a variation in the radiosensitivity within the two sub-groups [8–10] but overall, our data are in agreement with these studies as we clearly demonstrate that the two most radiosensitive of the four cell lines analysed in our study are HPV-positive. Two recent reports have implicated DSB repair deficiency in HPV-positive HNSCC which may be responsible for the observed increase in radiosensitivity [9, 12]. Specifically one report highlighted defects in both NHEJ and HR as demonstrated by reduced protein expression, and also foci formation post-IR of DNA-Pk and BRCA2, respectively [12]. This was shown in two HPV-positive HNSCC cells (UMSCC47 and UPCI-SCC154) versus one HPV-negative HNSCC cell line (UMSCC1). Therefore in order to corroborate these data, we examined the expression of key players involved in NHEJ and HR by quantitative Western blotting using extracts derived from the four OPSCC cell lines used in our study. We discovered that there was a significant reduction in the protein levels of Ku86, DNA-Pk, 53BP1 and BRCA2 in the UPCI-SCC090 HPV-positive OPSCC cell line versus the HPV-negative UMSCC6 and UMSCC74A cell lines (Figure 1C and 1D). This deficiency in DSB repair protein levels, and predictably in DSB repair, is consistent with the UPCI-SCC090 cells being the most radiosensitive (Figure 1B). In contrast, the levels of these proteins in the UMSCC47 HPV-positive OPSCC cells were not significantly different from the HPV-negative cells (Figure 1C and 1D), although there was a significant reduction in RAD51.

**Figure 2:**
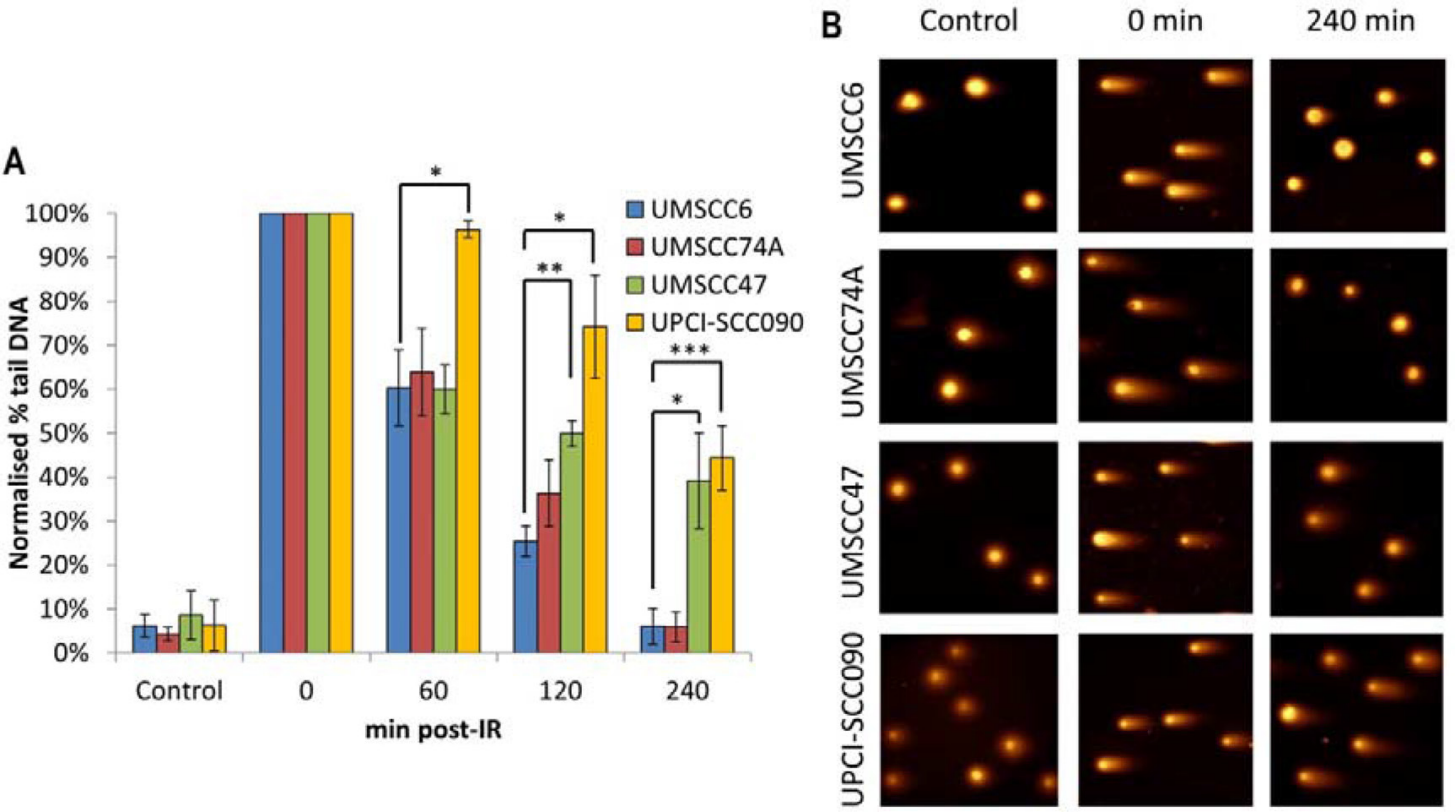
Comparative efficiency of the repair of DNA DSBs in HPV-negative and HPV-positive OPSCC cells (**A**) Cells were irradiated (4 Gy) and DNA DSBs measured at various time points post-IR by the neutral single cell gel electrophoresis (comet) assay. Shown is the % tail DNA with standard deviations from at least three independent experiments normalised to the levels seen immediately post-IR (0 min) which was set to 100 %. *p < 0.05, **p < 0.01, ***p < 0.005 as analysed by a one sample *t*-test of normalised % tail DNA values in the respective cells relative to the UMSCC6 cells at each particular time point. (**B**) Representative images of OPSCC cells visualised by the neutral comet assay, demonstrating defective repair of DNA DSBs in HPV-positive versus HPV-negative OPSCC cells.

In order to directly examine the relative efficiency of the OPSCC cells in performing DSB repair, and their correlation with DSB repair protein levels, we monitored the kinetics of repair of IR-induced DNA DSBs using the neutral comet assay. We observed that both sets of cells displayed similar levels of DNA DSBs in untreated conditions, demonstrating that the baseline level of DSBs is similar in all the four OPSCC cell lines and is not greatly affected by HPV status (Figure 2A and 2B, see controls). Following IR and subsequent incubation to allow for DNA repair, the two HPV-negative cells (UMSCC6 and UMSCC74A) both show similar kinetics for the repair of DNA DSBs which appear to return to baseline levels within 4 hours (Figure 2A and 2B). In contrast, the two HPV-positive cells (UMSCC47 and UPCI-SCC090) show delayed DNA DSB repair kinetics, albeit with different profiles. The UMSCC47 cells retain similar levels of DNA DSBs to the HPV-negative cells within 1 h post-IR, although increased levels of DSBs are observed 2–4 h post-IR (Figure 2A and 2B). Consequently the UMSCC47 cells display impaired DSB repair. In contrast, the UPCI-SCC090 have increased DSB levels at all the time points investigated (1–4 h) post-IR in comparison to the HPV-negative cells. This demonstrates that the UPCI-SCC090 cells are defective in the rate of DSB repair, and which correlates with the significantly reduced levels of multiple DSB repair proteins involved in this repair pathway (Figure 1C and 1D). Consequently, although both HPV-positive OPSCC cells (UMSCC47 and UPCI-SCC090) used in this study display reduced rates of DSB repair, relative to the HPV-negative OPSCC cells, two different mechanisms appear to be responsible for these cellular effects.

### HPV-positive OPSCC (UMSCC47) cells display persistent IR-induced γH2AX and 53BP1 foci formation

**Figure 3:**
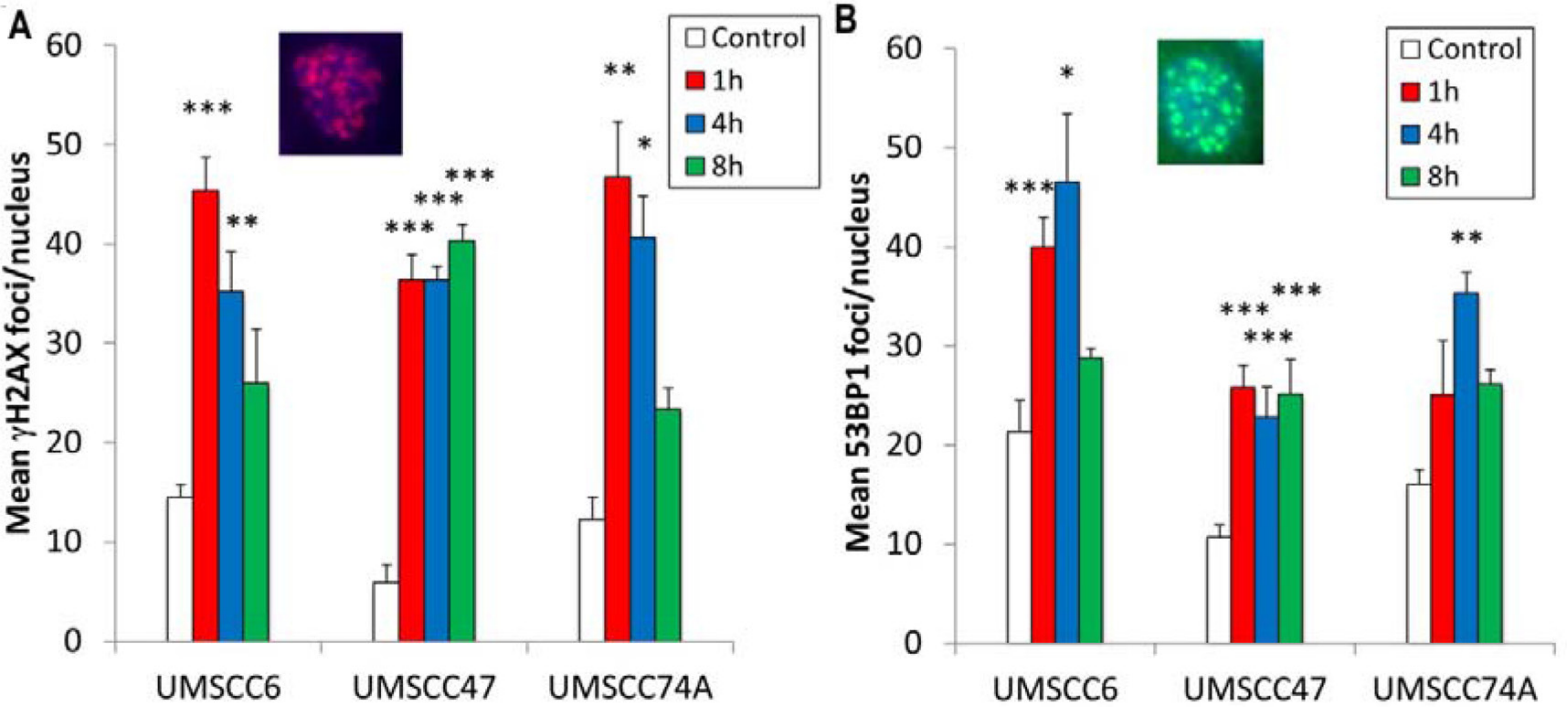
Analysis of IR-induced γH2AX and 53BP1 foci formation in HPV-negative and HPV-positive OPSCC cells OPSCC cells were irradiated (4 Gy) and (**A**) γH2AX and (**Β**) 53BP1 foci analysed by immunofluorescent staining at various time points post-IR. Shown is the mean number of foci/nucleus with standard errors from three independent experiments performed in triplicate. *p < 0.02, **p < 0.005, ***p < 0.001 as analysed by a one sample *t*-test of foci/nucleus at each particular time point compared to the respective untreated controls. Shown inset are immunofluorescence images showing nuclei with γH2AX or 53BP1 foci.

**Figure 4:**
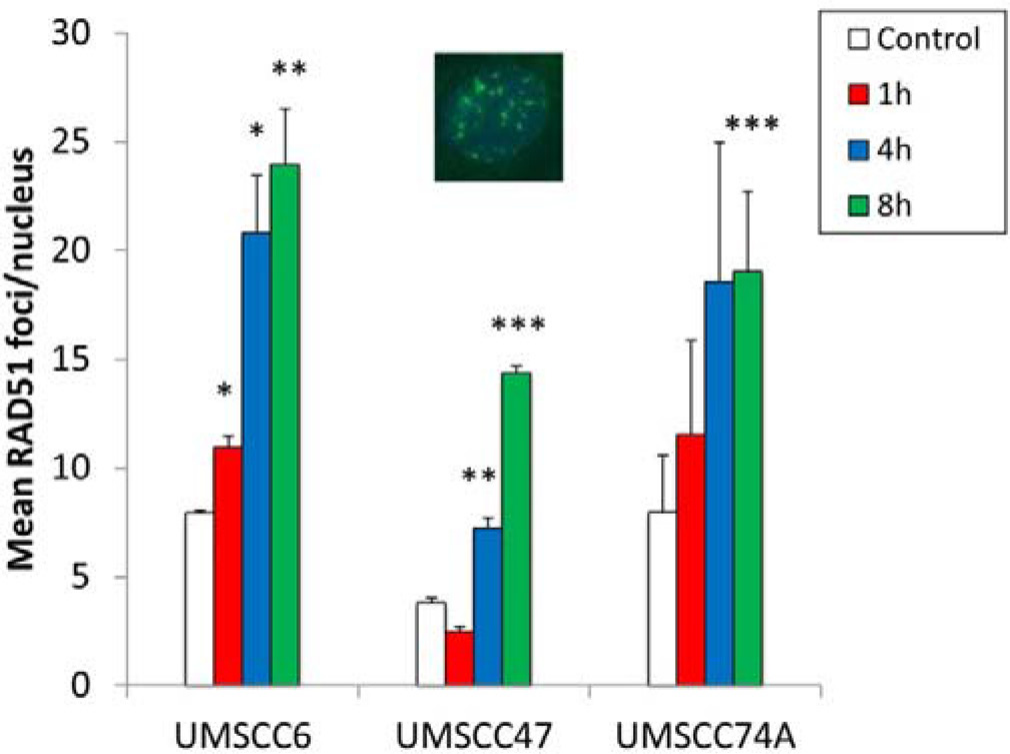
Analysis of IR-induced RAD51 foci formation in HPV-negative and HPV-positive OPSCC cells OPSCC cells were irradiated (4 Gy) and RAD51 foci analysed by immunofluorescent staining at various time points post-IR. Shown is the mean number of foci/nucleus with standard errors from two independent experiments performed in triplicate. *p < 0.02, **p < 0.01, ***p < 0.005 as analysed by a one sample *t*-test of foci/nucleus at each particular time point compared to the respective untreated controls. Shown inset is a representative immunofluorescence image showing nuclei with RAD51 foci.

Whilst we had confirmed defective DSB repair efficiency in HPV-positive OPSCC cells in comparison to HPV-negative OPSCC cells, we further examined the precise mechanism for deficiency in repair. This was of specific interest in relation to the UMSCC47 cells, which appeared to display relatively normal levels of DSB repair proteins, apart from a reduction in RAD51 (Figure 1C and 1D). Therefore we analysed the formation of both γH2AX and 53BP1 foci at various time points post-IR as markers of DSB recognition and of DSB processing through the predominant repair pathway NHEJ, respectively. We also analysed RAD51 foci as a marker of HR. This was performed in the UMSCC47 cell line, in comparison to the two HPV-negative cell lines, UMSCC6 and UMSCC74A. We observed that in UMSCC6 and UMSCC74A cells, γH2AX levels significantly increase ~3–4 fold at 1 h post-IR relative to the untreated controls (Figure 3A). The levels of γH2AX then start to decrease at 4 h post-IR, and at 8 h they are not significantly different from the untreated controls. Similarly, 53BP1 foci increase 1 h and 4 h post-IR in these cell lines, and then decrease at 8 h post-IR where they are similar to those seen in the untreated controls (Figure 3B). These kinetics of γH2AX and 53BP1 foci formation and disappearance are consistent with the efficiency of DSB repair, specifically via NHEJ. In the HPV-positive UMSCC47 cell line there is also a significant ~6-fold induction in γH2AX foci at 1 h post-IR relative to the untreated control, although in contrast to the UMSCC6 and UMSCC74A cells, γH2AX foci significantly persistent at 4 and 8 h post-IR (Figure 3A). Similarly, in the UMSCC47 cells there is an increase in 53BP1 foci 1 h post-IR and these also persist at 4 or 8 h post-IR (Figure 3B). This demonstrates that persistence of γH2AX formation, but also persistence of 53BP1 foci involved in NHEJ at the sites of DSBs, is further evidence of defective DSB repair in the HPV-positive UMSCC47 cell line. We next analysed RAD51 foci in response to IR. We observed a gradual increase in RAD51 foci, particularly at 4 h and 8 h post-IR, in both the HPV-negative UMSCC6 and UMSCC74A cells (Figure 4). Interestingly, despite the HPV-positive UMSCC47 cells containing significantly reduced protein levels of RAD51 (Figure 1C and 1D), these cells were also able to accumulate RAD51 foci at 4 h and 8 h post-IR which was significantly different from the untreated control (Figure 4). This suggests that these cells are competent in initiating HR.

### HPV-positive OPSCC cells have upregulated levels and activities of BER/SSB repair proteins

**Figure 5:**
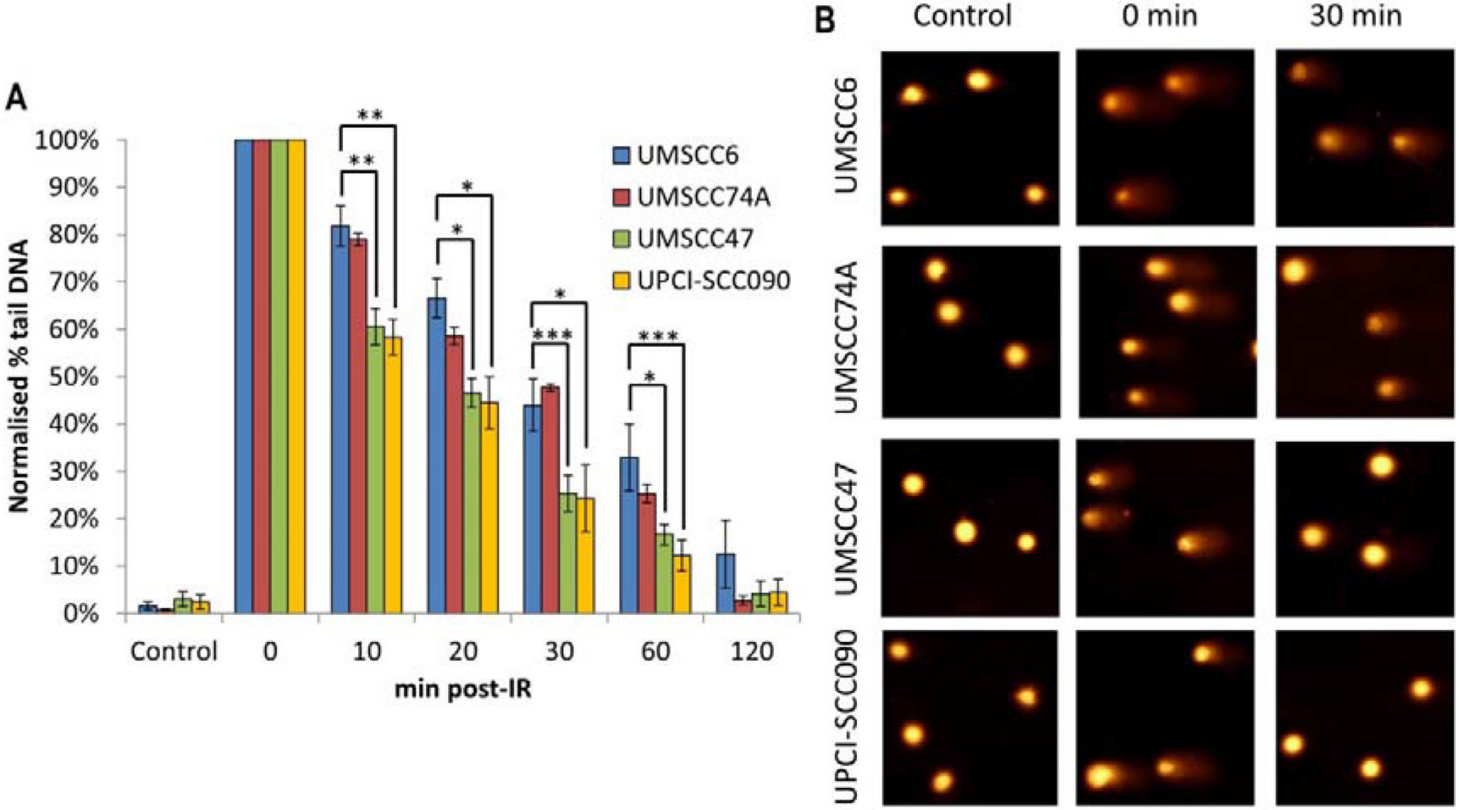
Comparative efficiency of the repair of DNA SSBs and alkali-labile sites in HPV-negative and HPV-positive OPSCC cells (**A**) Cells were irradiated (1.5 Gy) and DNA single strand breaks and alkali-labile sites measured at various time points post-IR by the alkaline single cell gel electrophoresis (comet) assay. Shown is the % tail DNA with standard deviations from at least three independent experiments normalised to the levels seen immediately post-IR (0 min) which was set to 100 %. (**B**) Representative images of OPSCC cells visualised by the alkaline comet assay, demonstrating faster repair kinetics of DNA SSBs/alkali-labile sites in HPV-positive versus HPV negative OPSCC cells. *p < 0.05, **p < 0.01, ***p < 0.002 as analysed by a one sample *t*-test of normalised % tail DNA values in the respective cells relative to the UMSCC6 cells at each particular time point.

**Figure 6:**
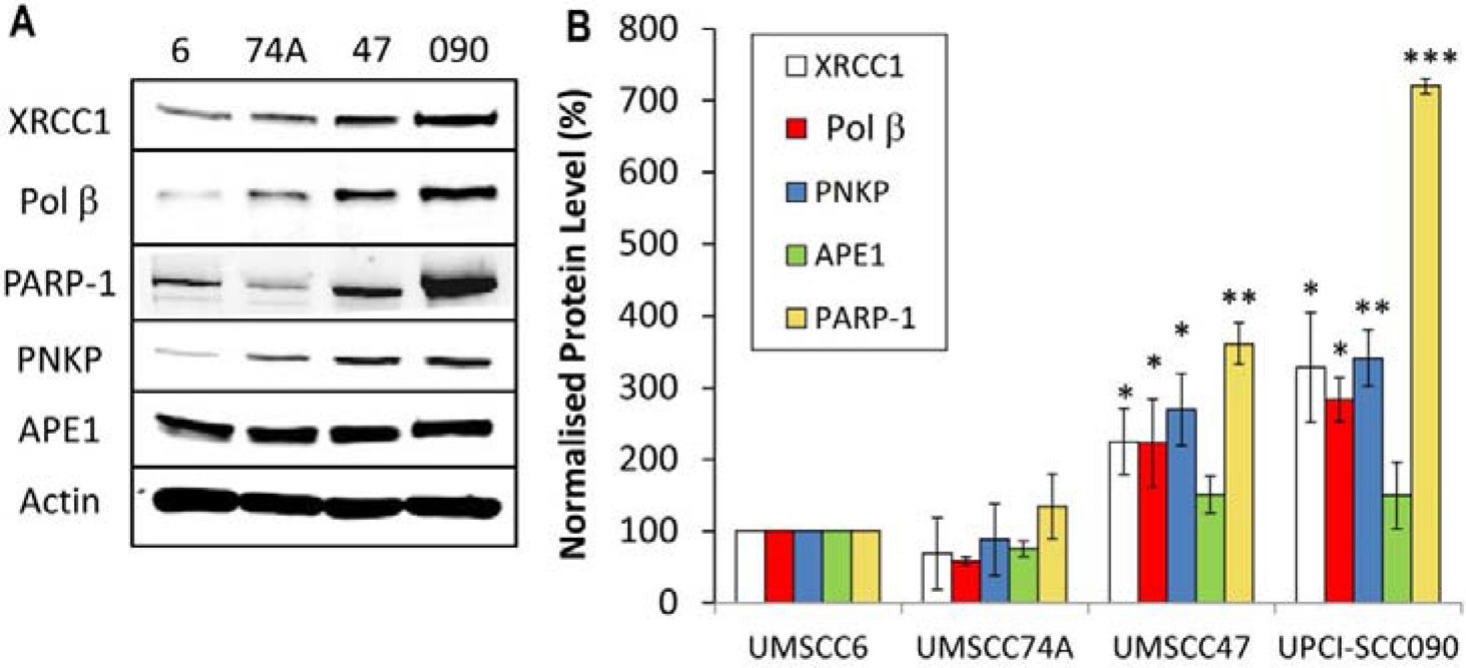
Analysis of BER and SSB repair protein levels in HPV-negative and HPV-positive OPSCC cells (**A**) Whole cell extracts from OPSCC cells were prepared and analysed by 10 % SDS-PAGE and immunoblotting with the indicated antibodies. (**B**) Levels of BER and SSB repair proteins relative to actin were quantified from at least three independent experiments. Shown is the mean protein level relative to actin with standard errors from at least three independent experiments, normalised to those calculated in the HPV-negative UMSCC6 cell extracts which was set to 100 %. *p < 0.05, **p < 0.01, ***p < 0.001 as analysed by a one sample *t*-test of normalised protein levels in the respective cell extracts relative to the UMSCC6 extracts.

Whilst we confirmed that HPV-positive OPSCC cells are defective in DSB repair which correlates with increased cellular radiosensitivity, since IR generates a high proportion of DNA base damage and SSBs which could also contribute to this phenotype, we analysed BER/SSB repair using the alkaline comet assay. Similar to results analysing DSBs, the baseline levels of SSBs and alkali-labile sites in all the cell lines tested was not significantly different (Figure 5A and 5B, see controls). In response to IR, both the HPV-negative OPSCC cells (UMSCC6 and UMSCC74A) show similar kinetics of repair of SSBs and alkali-labile sites as these gradually decrease from 10–60 min post-IR and return to approximately the levels seen in the untreated control at 2 h post-IR (Figure 5A and 5B). Surprisingly, we discovered that the HPV-positive OPSCC cells (UMSCC47 and UPCI-SCC090) display increased repair kinetics of SSBs and alkali-labile sites, as there are statistically significantly reduced levels of this DNA damage in these cells compared to the HPV-negative cells at 10–60 min post-IR (Figure 5A and 5B). This suggests that the levels and/or activities of proteins involved in BER/SSB repair are elevated in the HPV-positive OPSCC cells. To examine this in more detail, we analysed the levels of key BER/SSB repair proteins by quantitative Western blotting. We discovered that the levels of enzymes involved downstream in the BER/SSB repair pathway, namely Pol β and XRCC1 involved in gap filling and nick sealing, respectively were significantly higher in HPV-positive OPSCC cell lines (UMSCC47 and UPCI-SCC090) in comparison to HPV-negative OPSCC cells (UMSCC6 and UMSCC74A; Figure 6A and 6B). Furthermore the levels of PARP-1 and PNKP, involved in DNA strand break binding and processing, respectively were also significantly higher in the HPV-positive OPSCC cells. In contrast the proteins levels of APE1 (and actin as a loading control) were not significantly different in all of the four cell extracts (Figure 6A and 6B). This suggests that there is an upregulation of the BER/SSBR pathway in HPV-positive versus HPV-negative OPSCC cells, as evidenced by increased protein levels of key BER proteins and overall BER activity, which appears to inversely correlate with the DSB repair efficiency of the HPV-positive OPSCC cells.

In order to further understand the mechanism of altered BER protein expression in HPV-positive OPSCC cells, we analysed mRNA expression levels of key BER genes using quantitative PCR in comparison to mRNA derived from HPV-negative OPSCC cells, to examine whether this was caused by increases in DNA transcription. On comparison with the HPV-negative UMSCC6 OPSCC cell line, we did not find any significant elevation in the mRNA levels of Pol β, XRCC1, PARP-1 or PNKP (normalised against 18S ribosomal RNA housekeeping gene) in the HPV-positive cells, UMSCC47 and UPCI-SCC090 (Table 1). In fact the mRNA levels of these BER genes were even reduced in the UPCI-SCC090 cells. This suggests that increased DNA transcription is not responsible for the elevated BER protein levels observed in HPV-positive OPSCC cells, and most likely demonstrates that these changes occur at the protein level through increased protein stability and/or decreased protein degradation.

**Table 1:** mRNA expression levels of BER genes in OPSCC cells

	Fold change vs UMSCC6	
	UMSCC47	UPCI-SCC090
XRCC1	1.18 ± 0.79	0.52 ± 0.37*
Pol β	1.45 ± 1.39	0.76 ± 0.74
PNKP	1.34 ± 1.21	0.27 ± 0.14**
PARP-1	1.06 ± 0.94	0.53 ± 0.32*

Mean fold change with standard deviations from at least three independent experiments. *p < 0.02, **p < 0.001 as analysed by a one sample *t*-test.

### Sensitivity of HPV-positive and HPV-negative OPSCC cells to MMS and radiosensitisation by the PARP inhibitor olaparib

**Figure 7:**
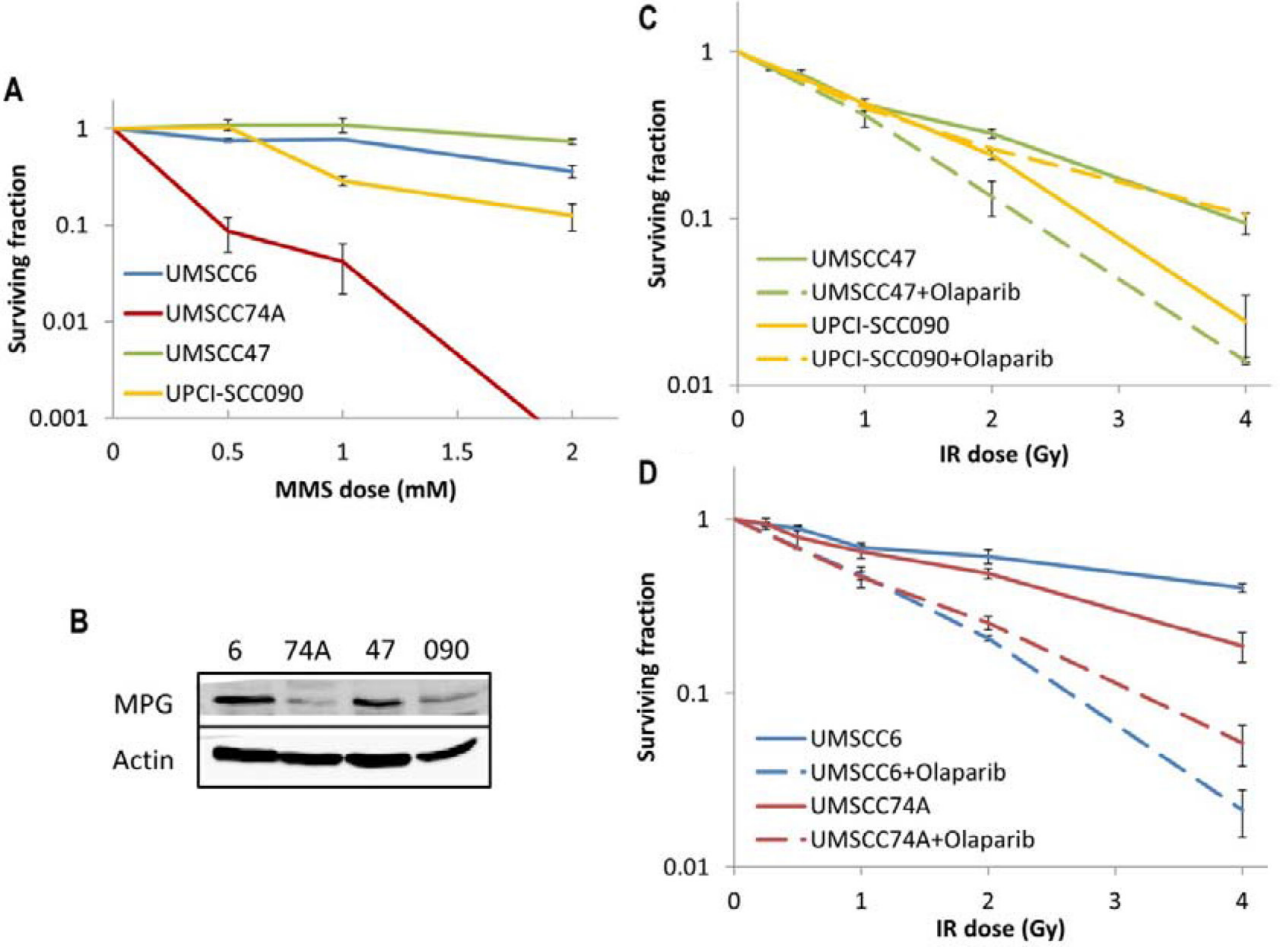
Analysis of MMS sensitivity and PARP inhibition on the radiosensitivity of HPV-negative and HPV-positive OPSCC cells (**A**) Clonogenic survival of OPSCC cells was analysed following treatment with increasing doses of MMS (0–2 mM). Shown is the surviving fraction with standard errors from at least three independent experiments. (**B**) Whole cell extracts from OPSCC cells were prepared and analysed by 10 % SDS-PAGE and immunoblotting with either MPG or actin antibodies. Clonogenic survival of (**C**) HPV-positive OPSCC cells or (**D**) HPV-negative OPSCC cells was analysed following treatment with increasing doses of IR (0–4 Gy) in the absence and presence of the PARP inhibitor olaparib (0.1 μM). Shown is the surviving fraction with standard errors from at least three independent experiments.

Since we discovered that HPV-positive OPSCC cells display a defect in DSB repair (Figure 2A), but have elevated protein levels and efficiency of BER (Figures 5A and 6A) in comparison to HPV-negative OPSCC cells, we examined whether this could be explored therapeutically. This is of particular importance in the HPV-negative OPSCC cells, which are relatively radioresistant. Therefore we investigated the effect of either an alkylating agent (methylmethanesulfonate; MMS) which generates DNA damage processed through BER, or the effect of a PARP inhibitor (olaparib) in combination with IR in causing effective cell killing. Interestingly, we found that one of the HPV-negative OPSCC cells (UMSCC74A) was extremely sensitive to MMS. We also discovered that the HPV-positive cell line (UPCI-SCC090) was mildly sensitive to MMS-induced cell kill whereas the UMSCC6 and UMSCC47 cells were relatively resistant (Figure 7A). In order to investigate these results further, we examined the protein levels of the DNA glycosylase involved in excision of alkylated DNA base damage, methylpurine DNA glycosylase (MPG). Indeed, levels of MPG were significantly lower in both UMSCC74A and UPCI-SCC090 cells that displayed sensitivity to MMS (Figure 7B). The combination of reduced MPG and reduced levels of other BER proteins specifically in the HPV-negative UMSCC74A cells would suggest why these cells display hypersensitivity to MMS.

PARP inhibitors have previously been shown to display synthetic lethality in killing BRCA-deficient tumours which are defective in DSB repair through HR [28, 29], and are currently being used in clinical trials. Since we have demonstrated that HPV-positive OPSCC cells are deficient in DSB repair but have elevated levels of PARP-1, we analysed whether the PARP inhibitor olaparib would sensitise these, and the HPV-negative OPSCC cells, to IR. We firstly demonstrated that olaparib (0.1 μM) was effective in suppressing IR-induced poly(ADP-ribose) polymer (PAR) formation in OPSCC cells, even in the UPCI-SCC090 cells that contain high protein levels of PARP-1 (Supplementary Figure 1A), and that olaparib alone (up to 1 μM) does not significantly affect survival of any of the cell lines (Supplementary Figure 1B). We discovered that only one of the HPV-positive OPSCC cells (UMSCC47) showed some increased IR sensitivity following PARP inhibition (dose enhancement ratios calculated at 50 % survival, DER = 1.51), whereas there was largely no effect on UPCI-SCC090 cells although these cells were the most radiosensitive of the four cell lines used (Figure 7C). Interestingly, olaparib had a more dramatic effect on the radiosensitisation of the HPV-negative OPSCC cell lines (Figure 7D). Indeed DER values of 3.34 and 1.79 for UMSCC6 and UMSCC74A, respectively were calculated. Therefore our data would suggest that PARP inhibition could be used therapeutically to target and radiosensitise HPV-negative, and to some extent HPV-positive, forms of HNSCC.

## DISCUSSION

The last three decades has seen a rapid rise in the incidence of HPV-positive OPSCC, and interestingly, patients with this disease display increased sensitivity to radiotherapy and chemotherapy and thus have improved survival rates in comparison to their HPV-negative counterparts [3–7]. Differential radiosensitivity is also recapitulated in cell lines derived from patients with HPV-positive and HPV-negative OPSCC [8–10], however the underlying cellular mechanisms responsible for this phenotype are unclear. Recently, it has been proposed that HPV-positive HNSCC cells are more radiosensitive than HPV-negative cells due to an impairment in the repair of DNA DSBs [9, 12]. A persistence of IR-induced DSBs measured via γH2AX/53BP1 foci was discovered in one report [9], whereas more conclusively γH2AX foci as well as IR-induced DSBs measured directly by neutral comet assays (albeit in only one HPV-positive HNSCC cell line) were shown to persist in HPV-positive versus HPV-negative HNSCC cells [12]. The precise mechanistic details responsible for defective DSB repair are still unclear as both reduced protein expression of 53BP1 and DNA-Pk involved in NHEJ, and BRCA2 and RAD51 involved in HR have been shown in two HPV-positive HNSCC cells, but only in comparison to a single HPV-negative cell line [12]. Furthermore, defective recruitment of DNA-Pk, BRCA2 and RAD51, but not 53BP1, to IR-induced DNA repair foci was found in the same study.

Our data using specifically OPSCC cells support the accumulating evidence demonstrating that DSB repair, measured directly by neutral comet assays, is indeed defective in HPV-positive cells versus HPV-negative cells, which correlates with increased cellular radiosensitivity. We also observed persistence of γH2AX and 53BP1 foci in the HPV-positive OPSCC cell line (specifically in UMSCC47 cells) post-IR, which corroborates previously published data suggesting that that these cells are defective in NHEJ [12]. However contrary to this previously published report, the exact mechanism for defective NHEJ in the two HPV-positive OPSCC cells used in our study are different. One cell line (UPCI-SCC090) showed a clear and significant reduction in the levels of key DSB repair proteins, including 53BP1, DNA-Pk and BRCA2 but also Ku86, which would support previous evidence, albeit using UPCI-SCC154 cells [12]. However, we were unable to show any reduced expression of these DSB repair proteins in the HPV-positive UMSCC47 cells, which the previous study had reported albeit non-quantitatively. Nevertheless, we are confident in our data as these have been validated by quantitative Western blotting using multiple extracts prepared from the cells at different times. It should also be noted that the two HPV-negative OPSCC cell lines used in our study are wild type p53 proficient, whereas the single cell line used in the previous study (UMSCC1; [12]) has been found to lack any detectable p53 [30]. We did however discover that the HPV-positive UMSCC47 cells contain reduced levels of RAD51 in comparison to the two HPV-negative OPSCC cell lines, but that the accumulation of IR-induced RAD51 foci was not impaired in these cells which again contradicts the previous study [12]. Collectively, our data highlight that DSB repair specifically through NHEJ is the major defect in HPV-positive OPSCC cells.

Interestingly, we have now discovered that HPV-positive OPSCC cells have quantitatively upregulated levels and activities of proteins involved in BER and SSB repair, including XRCC1, Pol β, PNKP and PARP-1. To our knowledge, this is the first demonstration for an association of HPV infection in OPSCC with modulation of BER protein levels, albeit in only two cell lines, and is therefore a novel finding. However there are reports that in general that HNSCC patients can have upregulated protein expression of BER proteins including XRCC1 [25], APE1 [22] and PARP-1 [27]. Previously, it has been reported that the HPV E6 protein can bind XRCC1, interfere with BER efficiency and increase sensitivity to MMS [31]. Although we found no evidence for a deficiency in the repair of IR-induced SSBs and alkali-labile sites in HPV-positive cells shown to contain higher levels of XRCC1, and in fact we uniquely discovered that BER activities were significantly increased. The reason for the upregulation of the BER pathway in these cells is unclear, although interesting given that they are defective in DSB repair. We were able to demonstrate that the mRNA levels of BER genes in HPV-positive OPSCC cells, are not significantly increased. Therefore the increased BER protein levels are not as a consequence of increased transcription, but appear to be modulated through increased protein stability or decreased protein degradation. Indeed we and others have demonstrated that BER proteins are targeted for ubiquitylation and subsequent degradation by the proteasome [14, 32]. Whether HPV infection directly interferes with ubiquitylation-dependent degradation of BER proteins, will be the subject of further investigation.

Due to our novel and interesting finding that BER proteins and activities are upregulated in two HPV-positive versus two HPV-negative OPSCC cells, we therefore investigated whether this repair pathway could be targeted therapeutically, particularly in HPV-negative OPSCC cells which are relatively radioresistant. Alkylating agents, such as MMS which generates DNA base alkylation predominantly repaired by the BER pathway, are a class of chemotherapy compounds used to treat cancers. We discovered that one HPV-negative OPSCC cell line (UMSCC74A) was extremely sensitive to the cell killing effects of MMS, whereas the other (UMSCC6) was mildly sensitive. Furthermore, one of the HPV-positive OPSCC cells (UPCI-SCC090) showed sensitivity intermediate to the two HPV-negative cells. We further discovered that this sensitivity to MMS was largely dependent on the levels of the DNA glycosylase involved in removal of DNA base alkylation, MPG. However BER proteins levels are also an important determinant in MMS sensitivity as the most sensitive cells were UMSCC74A, which contained reduced levels of both MPG and downstream BER proteins. Secondly, we investigated PARP inhibition, which is now an increasingly common strategy for cancer treatment particularly in BRCA-deficient tumours that are inefficient in HR [28, 29]. A previous study had shown increased radiosensitisation of HPV-positive HNSCC, including UMSCC47, to the PARP inhibitor veliparib [12]. Our data using the PARP inhibitor olaparib in combination with UMSCC47 cells are in good agreement with these data with similar DERs being obtained. In contrast, the other HPV-positive OPSCC cell line used in our study (UCPI-SCC090) showed no increased radiosensitisation with olaparib, although this cell line was the most radiosensitive of the four used. More importantly, the single HPV-negative HNSCC cell line (UMSCC1) used in this previous study [12] did not appear to be significantly radiosensitised on incubation with veliparib. This is in stark contrast to the data reported in our study where we have interestingly found that olaparib greatly radiosensitises two HPV-negative OPSCC cells (UMSCC74A and UMSCC6) that are proficient in DSB repair. Whilst the mechanism of radiosensitisation requires further investigation, it is again noteworthy here that UMSCC1 cells have been found not to express any significant levels of p53 [30], whereas the UMSCC74A and UMSCC6 cells express wild type p53, which may account for the observed differences. Nevertheless, our data would support the use of PARP inhibitors in combination with radiotherapy for the treatment of HPV-positive OPSCC, but particularly for sensitising radioresistant HPV-negative OPSCC. Therefore whilst alkylating agents, or PARP inhibitors in combination with IR appear to show promising results in our limited study using two HPV-negative OPSCC cell lines, we acknowledge that further studies are warranted to investigate this in more detail using a larger cohort of cells, but also in *in vivo* models.

## MATERIALS AND METHODS

### Materials

OPSCC cells (UMSCC6, UMSCC74, UMSCC47) were kindly provided by Prof T. Carey, University of Michigan, USA and were cultured in Dulbecco's Modified Eagle Medium (DMEM) supplemented with 15 % fetal bovine serum, 2 mM L-glutamine, 1× penicillin-streptomycin and 1× non-essential amino acids. UPCI-SCC090 were kindly provided by Dr S. Gollin from the University of Pittsburgh and were cultured in Minimal Essential Medium (MEM) supplemented with 15 % fetal bovine serum, 2 mM L-glutamine, 1× penicillin-streptomycin and 1× non-essential amino acids. All cells were cultured under standard conditions in 5 % CO_2_ at 37°C, and were authenticated in our laboratory by STR profiling. APE1, XRCC1, Pol β and PARP-1 antibodies raised in rabbit and affinity purified were kindly provided by Dr G. Dianov. PNKP antibodies raised in rabbit were kindly provided by Dr M. Weinfeld. DNA-Pk, RAD51, BRCA2, Ku-86 and PAR antibodies were from Santa Cruz Biotechnology (Heidelberg, Germany), 53BP1 antibodies were from Bethyl Laboratories (Montgomery, USA), p16 antibodies were from BD Biosciences (Oxford, UK) and γH2AX antibodies were from Millipore (Watford, UK). Actin antibodies were from Sigma-Aldrich (Gillingham, UK). Goat anti-mouse Alexa Fluor 555 and goat anti-rabbit Alexa Fluor 488 secondary antibodies for immunofluorescence staining were from Life Technologies (Paisley, UK).

### Clonogenic assays

Cells were harvested and a defined number seeded in triplicate into 6-well plates before being incubated overnight in 5 % CO_2_ at 37°C allowing for the cells to attach. For experiments involving PARP-1 inhibition, cells were also treated with olaparib (0.1 μM; Selleckchem, Munich, Germany) overnight, after allowing the cells to adhere for ~6 h. Cells were then irradiated with up to 4 Gy using a CellRad x-ray irradiator (Faxitron Bioptics, Tucson, USA) or treated with up to 2 mM MMS for 60 min in medium. Following treatment, fresh media was added to the cells and then colonies allowed to grow for 7–14 days, prior to fixing and staining with 6 % glutaraldehyde and 0.5 % crystal violet for 30 min. Plates were washed, left to air dry overnight and colonies counted using the GelCount colony analyser (Oxford Optronics, Oxford, UK). Relative colony formation (surviving fraction) was expressed as colonies per treatment level versus colonies that appeared in the untreated control.

### Whole cell extracts

Cells were grown in 10 cm dishes until ~70–80 % confluent, harvested and pelleted by centrifugation at 1500 rpm for 5 min at 4°C. Whole cell extracts were prepared from the cell pellets as previously described [33, 34]. Briefly, the pellets were resuspended in one packed cell volume (PCV) of buffer containing 10 mM Tris-HCl (pH 7.8), 200 mM KCl, 1 μg/ml of each protease inhibitor (pepstatin, aprotinin, chymostatin and leupeptin), 1 mM PMSF and 1 mM DTT. To the cell suspension, two PCV of buffer containing 10 mM Tris-HCl (pH 7.8), 600 mM KCl, 40 % glycerol, 0.1 mM EDTA, 0.2 % Nonidet P-40, 1 μg/ml of each protease inhibitor (pepstatin, aprotinin, chymostatin and leupeptin), 1 mM PMSF and 1 mM DTT was added and mixed thoroughly. The total cell suspension was mixed by rotation for 30 min at 4°C, the cell lysate centrifuged at 40,000 rpm for 20 min at 4°C and the supernatant collected, aliquoted and stored at −80°C.

### Western Blotting

Protein extracts (40 μg) were separated by 10 % or 6 % Tris-glycine SDS-PAGE, for medium and high molecular weight proteins, respectively and proteins transferred onto an Immobilon FL PVDF membrane (Millipore, Watford, UK). Membranes were blocked using Odyssey blocking buffer (Li-cor Biosciences, Cambridge, UK) and incubated with the primary antibody overnight at 4°C. Membranes were washed with PBS containing 0.1 % Tween 20, incubated with either Alexa Fluor 680 or IR Dye 800 secondary antibodies for 1 h at room temperature and further washed with PBS containing 0.1 % Tween 20. Proteins were visualized and quantified using the Odyssey image analysis system (Li-cor Biosciences, Cambridge, UK).

### RT-PCR analysis

Cells were grown in 10 cm dishes until ~70–80 % confluent, harvested and pelleted by centrifugation at 1500 rpm for 5 min at 4°C. RNA was prepared from cell pellets using the RNeasy kit (Qiagen, Crawley, UK) and cDNA was subsequently generated using the GoScript reverse transcription kit (Promega, Southampton, UK). Quantitative PCR (qPCR) reactions containing SYBR Select Master Mix (Life Technologies, Paisley, UK) and the following gene-specific primer pairs were prepared: 5′-AGG-TGC-AGA-GTC-CAG-TGG-TGA-3′ and 5′-GTC-AAG-CTG-GGA-TGG-GTC-AG-3′ (Pol β); 5′-CTG-GGA-CCG-GGT-CAA-AAT-3′ and 5′-CAA-GCC- AAA-GGG-GGA-GTC-3′ (XRCC1); 5′-GAT-CCT-GAG- AAC-CGG-ACA-G-3′ and 5′-CCC-GGT-AGT-TGA- GGG-GTT-3′ (PNKP); 5′-TCT-TTG-ATG-TGG-AAA- GTA-TGA-AGA-A and GGC-ATC-TTC-TGA-AGG-TCG-AT-3′ (PARP-1); 5′-GCA-ATT-ATT-CCC-CAT-GAA-CG and GGG-ACT-TAA-TCA-ACG-CAA-GC-3′ (18S rRNA). Reactions were analysed using the Applied Biosystems 7500 Real-Time PCR System (Life Technologies, Paisley, UK). Delta Ct values were calculated by subtracting Ct values for the gene of interest versus Ct values for the internal control (18S rRNA). Delta delta Ct values were generated by subtracting delta Ct values from the HPV-positive (UMSCC47 and UPCI-SCC090) cells versus the HPV-negative (UMSCC6) cells, and fold changes (2^ΔΔCt^) were calculated. For semi-quantitative PCR reactions and for confirming HPV status, cDNA was mixed with the following gene specific primer pairs: 5′-TTA-CCA-CAG-TTA-TGC-ACA-GA-3′ and 5′-ACA-GTC-GCT-TTT-GAC-AGT-TA-3′ (E6); 5′-ACA-GTC-GCT-TTT-GAC-AGT-TA-3′ and 5′-AGA-AAC-CCA-GCT-GTA-ATC-AT-3′ (E7). Products were amplified by PCR, separated by 2 % agarose gel electrophoresis in the presence of SYTO-60 (Life Technologies, Paisley, UK) and visualised using the Odyssey image analysis system (Li-cor Biosciences, Cambridge, UK).

### Single cell gel electrophoresis (comet) assays

The alkaline comet assay for measurement of DNA single strand breaks and alkali-labile sites was performed as recently described [35]. Briefly cells were trypsinised, diluted to 1 × 10^5^ cells/ml and 250 μl aliquots of the cell suspension placed into the wells of a 24 well plate placed on ice. Cells were irradiated in suspension at 1.5 Gy using a CellRad x-ray irradiator (Faxitron Bioptics, Tucson, USA) and embedded on a microscope slide in low melting agarose (Bio-Rad, Hemel Hempstead, UK). The slides were incubated for up to 2 h in a humidified chamber at 37°C to allow for DNA repair, prior to lysis in buffer containing 2.5 M NaCl, 100 mM EDTA, 10 mM Tris-HCl pH 10.5, 1 % (v/v) DMSO and 1 % (v/v) Triton X-100 for 1 h at 4°C. The slides were then incubated in the dark for 30 min in cold electrophoresis buffer (300 mM NaOH, 1 mM EDTA, 1 % (v/v) DMSO, pH 13) to allow the DNA to unwind, prior to electrophoresis at 25 V, 300 mA for 25 min. Slides were neutralised with three 5 min washes of 0.5 M Tris-HCl (pH 8.0) and allowed to air dry overnight. The neutral comet assay for measurement of DNA double strand breaks was similar to that described above, but with the following modifications. Cells were irradiated with 4 Gy x-rays and slides were incubated for up to 4 h to allow for DNA repair. Cell lysis was performed in buffer containing 2.5 M NaCl, 100 mM EDTA, 10 mM Tris-HCl pH 10.5, 1 % N-lauroylsarcosine, 1 % DMSO and 1 % (v/v) Triton X-100. Electrophoresis was performed in cold buffer containing 1 × TBE, pH 9.5 at 25 V, ~20 mA for 25 min. Finally slides were washed three times with 1 × PBS before allowing to air dry overnight. Slides from both alkaline and neutral comets were subsequently rehydrated for 30 min in water (pH 8.0), stained for 30 min with SYBR Gold (Life Technologies, Paisley, UK) diluted 1:10,000 in water (pH 8.0) and again air dried overnight. Cells (50 per slide, in duplicate) were analysed from the dried slides using the Komet 6.0 image analysis software (Andor Technology, Belfast, Northern Ireland) and % tail DNA values averaged from at least three independent experiments.

### Immunofluorescent staining and DNA repair foci analysis

OPSCC cells were grown on 13 mm coverslips until ~70–80 % confluent, irradiated at 4 Gy and incubated for the required time in 5 % CO_2_ at 37°C to allow for DNA repair. Cells were washed with PBS at room temperature for 5 min before being fixed using 4 % paraformaldehyde for 10 min. Cells were permeabilised with 0.2 % Triton X-100 in PBS for 10 min, then washed three times with 0.1 % Tween-20 for 10 min. Coverslips were blocked to avoid non-specific staining via incubation with 2 % BSA for 30 min at room temperature on a rocking platform with either γH2AX, 53BP1 or RAD51 antibodies in 2 % BSA overnight at 4°C. Following three washes with PBS, coverslips were incubated with either goat anti-mouse Alexa Fluor 555 or goat anti-rabbit Alexa Fluor 488 secondary antibodies in 2% BSA for 1 h at room temperature in the dark. Finally samples were washed with PBS for 10 min on a rocking platform and mounted on a microscope slide using Fluoroshield containing DAPI (Sigma-Aldrich, Gillingham, UK). Cells were examined using an Olympus BX61 fluorescent microscope with a Photometrics CoolSNAP HQ2 CCD camera. MicroManager software was used to capture images (~500 images/cell line/antibody).

## SUPPLEMENTARY MATERIALS FIGURES AND TABLES


