# Supplementary material for: Misregulation of DNA damage repair pathways in HPV-positive head and neck squamous cell carcinoma contributes to cellular radiosensitivity
Source: Oncotarget. 2017 Mar 16;8(18):29963–75. doi: 10.18632/oncotarget.16265 (PMC5444717; doi:10.18632/oncotarget.16265)
Supplement: Supplementary file 1 [file oncotarget-08-29963-s001.pdf]

Misregulation of DNA damage repair pathways in HPV-positive head and neck squamous cell carcinoma contributes to cellular radiosensitivity

Supplementary Materials

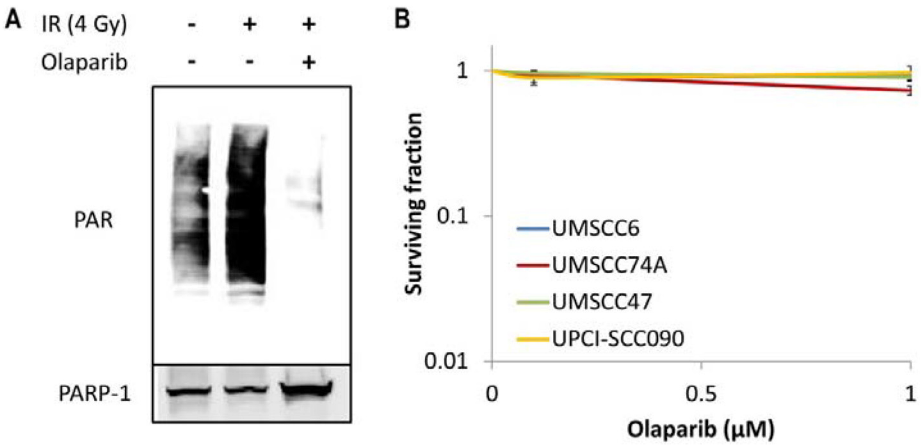

Supplementary Figure 1: Inhibition of PARP activity by olaparib in OPSCC cells and impact on cell survival. (**A**) UPCI-SCC090 cells were incubated in the absence or presence of olaparib (0.1 µM) for 24 h, either unirradiated or irradiated with x-rays (4 Gy) and harvested 15 min post-irradiation. Whole cell extracts were prepared and analysed by 10 % SDS-PAGE and immunoblotting with either antibodies raised against poly(ADP-ribose) polymers (PAR) or poly(ADP-ribose) polymerase (PARP-1). This demonstrates effective inhibition of PARP activity by olaparib post-irradiation. (**B**) OPSCC cells were incubated in the absence or presence of olaparib (0.1 µM and 1 µM) for 24 h prior to analysis of clonogenic survival. Shown is the surviving fraction with standard errors from at least three independent experiments. No significant decrease in cell survival was observed with olaparib compared to the untreated controls.
